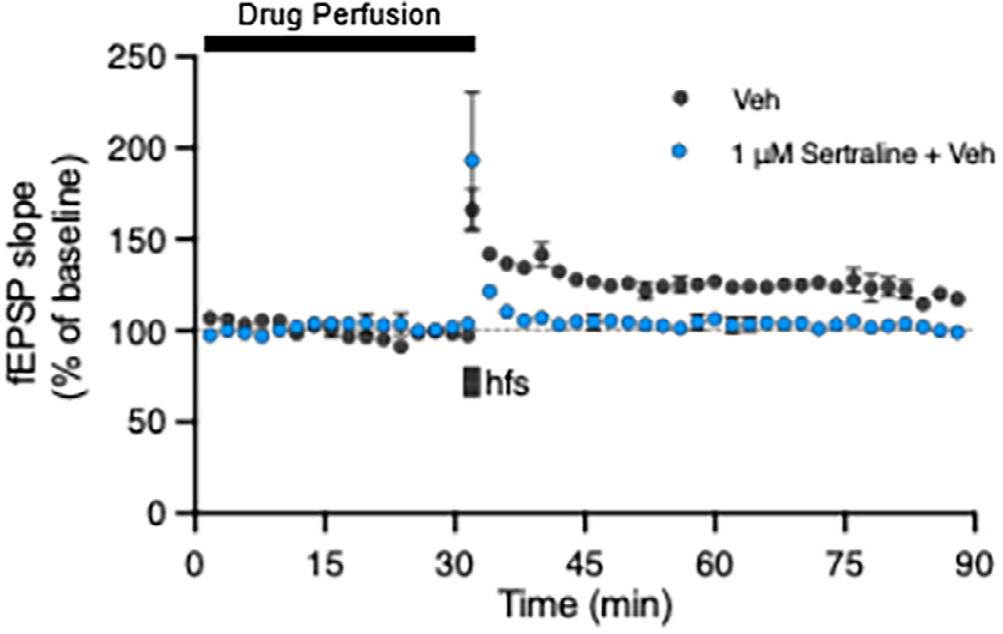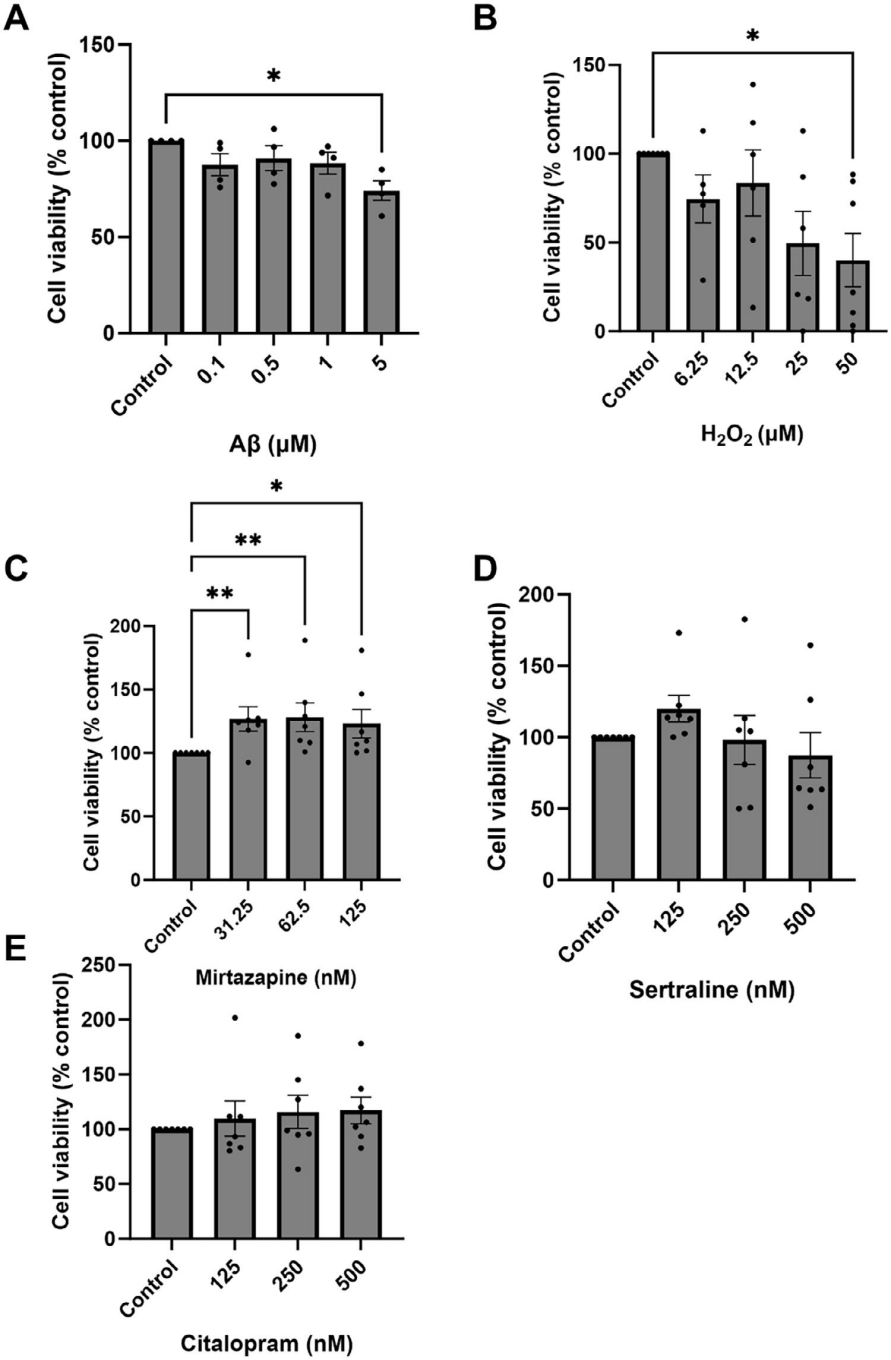# Potential neuroprotective effect of sertraline and mirtazapine on neuronal exposure to oxidative stress, amyloid beta and hypoxia

**DOI:** 10.1002/alz70855_104358

**Published:** 2025-12-24

**Authors:** Mizuki Morisaki, Oliver Milner, William W Watts, Daniel J Whitcomb, Robert MacLachlan, Robert A Fisher, Lucy A Crompton, Lindsey I Sinclair

**Affiliations:** ^1^ Cardiff university, Cardiff, Cardiff, United Kingdom; ^2^ University of Bristol, Bristol, United Kingdom; ^3^ University of the West of England, Bristol, United Kingdom

## Abstract

**Background:**

Although depression has been identified as a modifiable risk factor for Alzheimer's disease (AD) and other dementias it is not known whether this increase in risk is actually modifiable. We aimed to identify whether antidepressant medications show potential to mitigate this increase in risk using cell lines and acute rodent brain slices.

**Method:**

SH‐SY5Y cells differentiated into cholinergic neurons were pre‐treated with either antidepressants or vehicle control (DMSO) for 24h and primary human hippocampal neurons pre‐treated for 10 days prior to either hypoxia (2% O_2_ for 24h) or amyloid beta (1µM for 24/48h). Neuronal morphology was assessed using Holomonitor and synapse number by PSD‐95 staining. Acute hippocampal slices were obtained from juvenile P24 ‐ 30 male and female Wistar rats. Field recordings were obtained from the stratum radiatum of the CA1 region and evoked by 0.1 ms voltage pulses to either the Schaffer collateral (SC) pathway or subiculum.

Cell viability was assessed using the MTT assay, Mitochondrial number and morphology bystaining with mitored and mitochondrial health using TMRM. Long‐term potentiation (LTP) magnitude was calculated by comparing the post‐tetanus SC responses to baseline, with responses from the subiculum acting as a control input.

For all confocal imaging, ≥3 images were taken at random locations per well at x60 magnification and then analysis carried out in ImageJ. All other statistical analysis was performed in GraphPad Prism and R. We used a minimum of 3 repetitions for all cell line outcomes.

**Result:**

Hippocampal cell line data is still being analysed. Mirtazapine but not sertraline increased cell viability in the differentiated SH‐SY5Y neurons (*p* <0.05 125nM, *p* <0.01 62.5nM). Sertraline (*p* <0.001) reduced SH‐SY5Y mitochondrial numbers. Mitochondrial numbers increased following exposure to 1µM Aβ for 48h (sertraline *p* <0.001, mirtazapine *p* <0.001). Sertraline increased mitochondrial fragmentation (*p* <0.001) and mirtazapine increased mitochondrial elongation (*p* <0.001) after 1µM Aβ for 48h. Initial acute rodent slices experiments demonstrated that 1µM sertraline inhibited long‐term potentiation. Repetition with 500nM sertraline and with mirtazapine is underway.

**Conclusion:**

Our preliminary results suggest that sertraline and mirtazapine may reduce dementia risk, possibly by reducing Aβ mitochondrial toxicity or Aβ mediated excitotoxicity.